# Optimizing women's health: pelvic floor considerations in sports and physical activity

**DOI:** 10.3389/fspor.2026.1707274

**Published:** 2026-02-02

**Authors:** Arianna Bortolami, Giacomo Rossettini

**Affiliations:** 1 Pelvic Floor Studio, Padova, Italy; 2 School of Physiotherapy, University of Verona, Verona, Italy

**Keywords:** clinical reasoning, decision making, evidence based, outcome, pain, physiotherapy, rehabilitation, sports

## 1 Introduction

The pelvic floor is a complex system of muscles and connective tissue that supports the genitourinary–anal region and plays a central role in urinary, anorectal, sexual, reproductive, and biomechanical functions. Pelvic floor dysfunctions (PFD) have gained increasing attention due to their substantial impact on women's quality of life (1). Across the female lifespan, PFD may be associated with physiological events such as pregnancy, childbirth (2), and menopause (3), as well as with gynecological disorders (e.g., endometriosis and vulvodynia) (4–6) and systemic conditions (e.g., fibromyalgia and scleroderma) (7, 8).

In recent years, certain forms of sports and physical activity have also been identified as factors that may influence pelvic floor function. However, research and clinical attention have largely focused on disorders associated with reduced pelvic floor muscle tone, particularly urinary incontinence, while other disorders associated with increased PFM tone, like pain, or disorders of PFM coordination, remain less explored (9–12).

This opinion paper aims to raise awareness of the role of sports and physical activity in women when PFD are not adequately considered, with particular emphasis on conditions characterized by increased pelvic floor muscle tone. Specifically, the paper addresses: (A) the classification of PFD and related symptoms; (B) current evidence on the relationship between pelvic floor and sports activity; (C) existing research gaps; and (D) the implications for clinical practice.

## 2 Discussion

### 2.1 Classification of PFD and correlated symptoms

PFD have traditionally been categorized into four broad groups: normal pelvic floor muscles (PFM), underactive PFM, overactive PFM, and non-functioning PFM (13). More recently, this framework has been refined to better reflect clinically relevant subgroups, including disorders of decreased PFM tone, increased PFM tone (e.g., pelvic floor tension myalgia, pelvic floor myofascial pain syndrome), PFM pain (e.g., pelvic floor myalgia), PFM coordination (e.g., dyssynergia as vaginismus and anismus), and pudendal neuralgia (14). In this paper, we adopt the classification proposed by Frawley et al., which is aligned with international scientific societies and provides a comprehensive framework for pelvic floor assessment (14). This classification allows clinicians to move beyond a simplified strength-based perspective and to capture the multidimensional nature of PFD.

Clinical manifestations vary according to the underlying condition. Disorders of decreased PFM tone are commonly associated with urinary incontinence, pelvic organ prolapse (15), bladder and rectal filling symptoms (16), and sexual dysfunction linked to reduced genital sensation and pleasure (17). In contrast, disorders characterized by increased PFM tone, pain, or impaired coordination are more frequently associated with chronic pelvic pain, sexual dysfunction, and difficulties with vaginal penetration (17, 18) These conditions are currently encompassed within the Diagnostic and Statistical Manual of Mental Disorders (DSM) as dyspareunia and vaginismus in DSM-IV (DSM Fourth Edition), or as genito-pelvic pain/penetration disorder in DSM-V (DSM Fifth Edition) (19). Additional symptoms may include impaired bladder or anorectal emptying, reflecting altered relaxation or coordination of the pelvic floor (16) as well as structural presentations such as obstructed defecation (20).

Overall, this classification highlights the heterogeneity of PFD and underscores the importance of accurately identifying the specific pelvic floor presentation when evaluating women engaged in sports or physical activity. A classification-driven assessment is essential to inform appropriate clinical reasoning and guide targeted therapeutic approaches (14).

### 2.2 What we already know

Regular and appropriately dosed sport and physical activity provide well-established health benefits across the female lifespan, contributing to the prevention and management of major non-communicable diseases and supporting physical and mental well-being (21–23). These benefits are evident during adolescence (24), adulthood (25), pregnancy and the postpartum period (26–28), and menopause (29).

Beyond systemic effects, exercise induces structural and functional adaptations in skeletal muscle (30). In women, some of these responses appear to be hormone-sensitive: estrogen receptors have been identified in skeletal muscle, particularly in type II fibers, and may contribute to protein synthesis and muscle repair following exercise (31). The menopausal decline in estrogen is associated with reductions in muscle mass and strength, contributing to sarcopenia (32). These observations highlight the importance of considering hormonal status and life stage when prescribing physical activity.

Sports and physical activity can directly influence PFM through increases in intra-abdominal pressure, activation of the core muscle system, transmission of ground-reaction forces, and upper-limb exertions. These include abdominal contractions that increase intra-abdominal pressure (9), activation of the core muscle system of which the pelvic floor is an integral component (33, 34), transmission of ground-reaction forces from the lower limbs during walking (33), running (35), or jumping (36), and upper-limb exertions, such as weightlifting, that further increase pelvic loading (37).

Given these interactions, pelvic floor health has been formally recognized as one of the ten domains of female athlete health that should be monitored within illness and injury surveillance frameworks (38). Nevertheless, the reported effects of sport and physical activity on PFM function remain heterogeneous. Some studies suggest negative effects on muscle performance (9), others indicate no clear effect (39), while several report improvements or no apparent harm (40). These discrepancies likely depend on the type and intensity of activity (41), individual factors and life events such as childbirth (10) or menopause (42), and the contribution of the pelvic floor and related muscles, such as the transvs. abdominis, in managing intra-abdominal pressure (43). Prior exposure to physical activity may also influence pelvic floor status at specific stages of life (40).

From a symptomatic perspective, urinary incontinence remains the most frequently investigated PFD in athletes, with consistently high prevalence reported across multiple sports (44). High rates of PFD symptoms have been described in disciplines such as rugby, powerlifting, trampolining, volleyball, basketball, and martial arts (45), as well as in female aerialists (46) and artistic athletes (47). Female runners with stress urinary incontinence have been shown to experience higher intra-abdominal pressure during running compared with continent runners (48). By contrast, evidence regarding other pelvic floor dysfunctions, including anal incontinence and pelvic organ prolapse, remains limited (11).

Despite growing research interest, awareness and help-seeking behaviors among female athletes remain suboptimal. Many women are reluctant to disclose PFD symptoms to healthcare professionals (49), and knowledge of the association between sport and pelvic floor health is generally low (50). Educational interventions may improve awareness and reduce symptoms (51), yet embarrassment and communication barriers persist, with negative consequences for performance and quality of life (52).

Encouraging evidence supports the effectiveness of treatment strategies for sport-related PFD. Pelvic floor muscle training has shown positive outcomes (43), although most studies focus primarily on urinary incontinence and on a limited range of sports, such as cycling, trampoline gymnastics, and other high-impact disciplines. Importantly, considering pelvic floor load during sports practice appears essential for appropriate management (53). Education alone may not always be sufficient to change behavior (54), though in some athlete groups increased awareness has been associated with reduced symptom prevalence (51).

Overall, current evidence supports the relevance of pelvic floor health in sports contexts but also highlights substantial heterogeneity and important limitations. Research has largely emphasized dysfunctions associated with reduced PFM tone, leaving other clinically relevant pelvic floor presentations comparatively underexplored.

### 2.3 Gaps in current research

Although the existing literature has advanced understanding of pelvic floor health in female athletes, substantial gaps remain. Research has focused predominantly on urinary incontinence, a condition commonly associated with decreased PFM tone. While this focus has improved knowledge of weakness-related dysfunctions, it has simultaneously limited investigation into other clinically relevant pelvic floor presentations.

In particular, disorders associated with increased PFM tone, PFM pain, disorders of PFM coordination, and pudendal neuralgia have received little investigation. Certain activities that emphasize sustained trunk stabilization and core activation, such as classical dance (55), yoga practices (56, 57), Pilates (58), Tai Chi (59), and abdominal exercises (60), may place specific and prolonged demands on the pelvic floor. As the pelvic floor is an integral component of the core muscle system, activation strategies commonly used in these disciplines may facilitate increased PFM activity.

Accordingly, it has often been assumed that women experiencing symptoms related to increased PFM tone, pain, or pelvic floor coordination may not benefit from further PFM activation. To date, this hypothesis is largely grounded in clinical reasoning rather than high-quality experimental evidence. However, this may primarily apply to women who do not undergo pelvic floor physiotherapy, given emerging evidence suggesting that PFM contraction can be used in selected contexts (61) and may not worsen genital pain; nonetheless, effects on symptoms related to increased tone (e.g., pain, dyspareunia, bladder and anal-rectal symptoms), pelvic floor condition, and quality of life remain uncertain (62). Other studies have considered approaches emphasizing relaxation and increased flexibility during pelvic floor training (63, 64). Regarding different pelvic floor exercise modalities using contraction and/or relaxation, some researchers have recently explored the comparison between different approaches (65, 66), highlighting the possible role of contraction.

Despite being clearly recognized, classified, and routinely addressed in physiotherapy practice (67, 68), disorders characterized by increased PFM tone remain underrepresented in sports-related pelvic floor research. These conditions may be under-recognized in physically active women, partly because their symptoms overlap with more frequently investigated presentations such as urinary incontinence or musculoskeletal pain. In sports contexts characterized by repetitive loading, high training volumes, and performance-driven demands, these phenotypes may remain clinically relevant despite limited direct evidence. Explicit consideration of increased PFM tone within differential diagnosis may therefore help avoid overly simplified interpretations of PFD in active women.

Another gap concerns the relationship between exercise and chronic pelvic pain. Although physical activity is recognized as beneficial in the management of chronic pain (69) and musculoskeletal disorders (70), evidence regarding its effects on pelvic-specific pain conditions remains scarce. It is unclear whether similar analgesic mechanisms apply when pelvic floor involvement is present.

Furthermore, the pelvic floor is not always adequately considered in the differential diagnosis of pain conditions commonly encountered in athletes. Pelvic floor dysfunction may contribute to symptoms attributed to hip and groin pain (71–74) or lumbopelvic disorders (75), yet it is often overlooked during musculoskeletal assessment. Clinicians managing athletes with persistent or unexplained pain should therefore consider the pelvic floor as a potential contributing factor and assess it accordingly (71).

From a clinical perspective, physiotherapists and other health professionals are frequently required to evaluate sports participation as either a potential risk factor or a therapeutic resource for PFD (9). Management strategies may include symptom surveillance, modification of training load or intensity, substitution of high-impact exercises, encouragement of appropriate physical activity in sedentary women, or temporary suspension of sport during symptom exacerbation. Collaboration with coaches and trainers may facilitate safe training adaptations, while pelvic floor–specific therapeutic exercises can support symptom management. Importantly, these interventions should be framed within a biopsychosocial approach that balances symptom control with the recognized benefits of sport for women's health and quality of life (9).

Additional gaps relate to the preventive role of physical activity and to the adaptation of sports participation across specific life stages. It remains unclear whether particular exercise modalities can reduce the risk of pelvic floor dysfunction in asymptomatic women, or how training should be modified during periods such as menopause, when hormonal and urogenital changes may alter pelvic floor function. Addressing these gaps is essential to inform individualized exercise prescriptions and improve clinical decision-making.

### 2.4 Implications for clinical practice and research

The limited and uneven evidence base on PFD in female athletes has direct implications for clinical practice (Table 1). Women engaged in competitive or recreational sports are often insufficiently informed that certain types of physical activity may be more likely to provoke or expose pelvic floor symptoms than others (51). As a result, preventive strategies are rarely implemented, and targeted interventions for symptomatic women are infrequently applied, increasing the likelihood of symptom progression.

**Table 1:** Clinical considerations for sports and physical activity in relation to pelvic floor health.

Overview of potential effects, clinical considerations, and screening related to sports and physical activity and pelvic floor health
Potential mechanisms by which sports and physical activity may influence pelvic floor function • Changes in muscle parameters (strength, endurance, power, relaxation, tone, trophism) • Increased intra-abdominal pressure • Recruitment of the “core” functional unit • Effect of heel strike during walking, running, and jumping • Effect of upper limb involvement Examples of individualized strategies that may be considered in clinical practice for women with pelvic floor dysfunctions • Active surveillance • Reduction in frequency • Reduction of loads • Replacement of selected exercises • Substitution with another activity • Initiation of sport activity in sedentary women • Temporary suspension of sport activity • Continuation of the current activity under monitoring • Communication with the coach/trainer • Pelvic floor therapeutic exercises immediately after sport activity • Increased therapeutic exercises during periods of intense activity Examples of screening questions for clinical and preventive practice: • “Do you practice sports or physical activity?” • “If not, why?” • “If yes, which sports or physical activities?” • “For how long?” • “How many times per week?” • “Have you practiced other sports before, and if so, which?” • “Have you engaged in competitive or high-impact sports?” • “How important are sports and physical activity for your quality of life/well-being?” • “Have you ever modified or stopped physical activity for health reasons involving the pelvic area?” • “If so, were these problems associated with urinary symptoms (e.g., involuntary urine leakage or urgency), anal-rectal symptoms (e.g., involuntary gas or stool leakage, constipation), sensations of heaviness or vaginal bulging, or genital pain?” • The PFD-SENTINEL tool may also be useful.

PFD-SENTINEL, Pelvic Floor Dysfunction-ScrEeNing Tool IN fEmale athLetes.

Health professionals face similar challenges. Physiotherapists and other clinicians involved in prevention and management of PFD are frequently required to determine whether sports participation should be considered a potential risk factor or a therapeutic resource (76). In the absence of robust evidence-based guidance, clinical decision-making often relies on individualized assessment, taking into account symptom presentation, overall health status, life stage, and sport-specific demands. This variability highlights the need for clinical frameworks that integrate pelvic floor health into sports medicine and rehabilitation.

Patient education and screening represent additional critical areas. Women should be informed about the relevance and potential benefits of pelvic floor screening before engaging in physical activity, such as sports or exercise. Effective screening requires validated tools when available, or alternatively the use of structured, clinically informed questioning supported by emerging evidence (77). However, currently available screening instruments remain limited (78). When formal tools are impractical, clinicians should explore women's physical activity history through targeted yet conversational assessment, including type, duration, frequency, and intensity of activity; exposure to high-impact or competitive sports; previous sports participation; and the perceived role of physical activity in quality of life and well-being (49).

From a research perspective, future studies should prioritize the investigation of sports and exercise modalities involving sustained or intense trunk stabilization in women presenting with disorders of increased PFM tone, pelvic floor pain, disorders of PFM coordination, or pudendal neuralgia. Further investigation is also needed to clarify whether sport can be safely and effectively used as a therapeutic strategy in chronic pelvic pain conditions. More broadly, research should aim to identify when sports participation represents a risk factor and when it may act as a protective or rehabilitative intervention, particularly across different stages of a woman's life, such as menopause, when hormonal and musculoskeletal changes may influence pelvic floor function. It may also be helpful to determine when physical activity has no meaningful consequences for PFD (e.g., neither beneficial nor harmful) because this can help remove barriers to exercise driven by currently unfounded claims.

**Figure 1:**
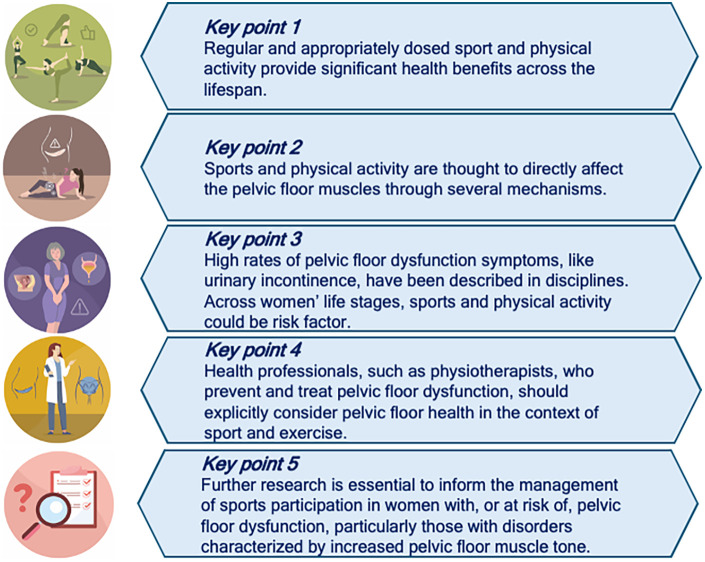
Correlations between pelvic floor and sports activity. PFM pelvic floor muscles, PFD pelvic floor dysfunction.

Addressing these clinical and research gaps will support the development of individualized, evidence-informed approaches that integrate sports participation into prevention and management pathways for PFD, while empowering women and professionals to make informed decisions that balance pelvic floor health with the well-established benefits of physical activity (Figure 1).

## 3 Conclusions

Sports and physical activity are fundamental for women's health across the lifespan, yet they may act as potential risk factors for provoking or exacerbating symptoms when PFD is already present. Sports professionals and healthcare providers should therefore explicitly consider pelvic floor health when designing and managing exercise programs.

Clinical management should be individualized, taking into account sport participation, overall health status, and life stage to maximize benefits while minimizing symptom exacerbation. Further research is needed to inform evidence-based management of sports participation in women with, or at risk of, PFD, and to clarify the balance between potential risks and therapeutic benefits across the female lifespan.
